# Thidiazuron combined with cyclanilide modulates hormone pathways and ROS systems in cotton, increasing defoliation at low temperatures

**DOI:** 10.3389/fpls.2024.1333816

**Published:** 2024-04-03

**Authors:** Hongmei Shu, Shangwen Sun, Xiaojing Wang, Jian Chen, Changqin Yang, Guowei Zhang, Huanyong Han, Zhikang Li, Ting Liang, Ruixian Liu

**Affiliations:** ^1^ Institute of Industrial Crops, Jiangsu Academy of Agricultural Sciences/Key Laboratory of Cotton and Rapeseed, Ministry of Agriculture and Rural Affairs, Nanjing, China; ^2^ Institute of Food Safety and Nutrition, Jiangsu Academy of Agricultural Sciences, Nanjing, China; ^3^ Cotton Research Institute, Xinjiang Academy of Agricultural and Reclamation Science, Shihezi, China

**Keywords:** cyclanilide, thidiazuron, cotton, low temperature, defoliation

## Abstract

Low temperatures decrease the thidiazuron (TDZ) defoliation efficiency in cotton, while cyclanilide (CYC) combined with TDZ can improve the defoliation efficiency at low temperatures, but the mechanism is unknown. This study analyzed the effect of exogenous TDZ and CYC application on cotton leaf abscissions at low temperatures (daily mean temperature: 15°C) using physiology and transcriptomic analysis. The results showed that compared with the TDZ treatment, TDZ combined with CYC accelerated cotton leaf abscission and increased the defoliation rate at low temperatures. The differentially expressed genes (DEGs) in cotton abscission zones (AZs) were subjected to Gene Ontology (GO) and Kyoto Encyclopedia of Genes and Genomes (KEGG) analyses to compare the enriched GO terms and KEGG pathways between the TDZ treatment and TDZ combined with CYC treatment. TDZ combined with CYC could induce more DEGs in cotton leaf AZs at low temperatures, and these DEGs were related to plant hormone and reactive oxygen species (ROS) pathways. CYC is an auxin transport inhibitor. TDZ combined with CYC not only downregulated more auxin response related genes but also upregulated more ethylene and jasmonic acid (JA) response related genes at low temperatures, and it decreased the indole-3-acetic acid (IAA) content and increased the JA and 1-aminocyclopropane-1-carboxylic acid (ACC) contents, which enhanced cotton defoliation. In addition, compared with the TDZ treatment alone, TDZ combined with CYC upregulated the expression of *respiratory burst oxidase homologs* (*RBOH*) genes and the hydrogen peroxide content in cotton AZs at low temperatures, which accelerated cotton defoliation. These results indicated that CYC enhanced the TDZ defoliation efficiency in cotton by adjusting hormone synthesis and response related pathways (including auxin, ethylene, and JA) and ROS production at low temperatures.

## 1 Introduction

The application of harvest aids is an important prerequisite for achieving mechanical cotton harvesting (*Gossypium hirsutum* L.). At present, thidiazuron (TDZ) is widely used to accelerate cotton leaf abscission (Xu et al., 2019). TDZ application at the proper time before cotton harvesting can promote leaf abscission, which can decrease the debris content and increase the fiber quality of machine-picked cotton (Siebert and Stewart, 2006; Du et al., 2014). However, low temperatures remarkably decrease the efficiency of TDZ-induced cotton leaf abscission (Snipes and Wills, 1994; Eder et al., 2017; Shu et al., 2022), which cannot meet the requirements for machine-picked cotton. Therefore, improving cotton defoliation efficiency at low temperatures has become an important challenge in the mechanized cotton harvesting process.

It was found that TDZ mainly regulated ethylene and auxin to activate abscission signaling in abscission zones (AZs), followed by activating cell wall hydrolases to promote middle lamella degradation and primary cell wall loosening of the separation layers (Xu et al., 2019; Li et al., 2022). However, low temperatures affect the biosynthesis and signaling gene responses of ethylene and auxin in AZs to TDZ, resulting in the genes encoding cell wall hydrolases not being activated (Shu et al., 2022). The alterations in these pathways are closely related to the inhibition of TDZ-induced leaf abscission at low temperatures. In addition, reactive oxygen species (ROS) also play critical roles in regulating plant organ abscission (Liao et al., 2016b). The accumulation of hydrogen peroxide (H_2_O_2_) has been implicated in AZ activation (Bar-Dror et al., 2011; Yang et al., 2015; Liao et al., 2016b). Continuous H_2_O_2_ production is involved in the regulation of cell wall-degrading enzymes in AZs (Sakamoto et al., 2008a; Sakamoto et al., 2008b). H_2_O_2_ generated from respiratory burst oxidase homologs (RBOH) contributes to TDZ-induced leaf abscission (Li et al., 2021).

Cyclanilide (CYC) is a plant growth regulator registered for use in cotton, but it is only used as an adjuvant to enhance other growth regulator activities (Burtom et al., 2008). CYC itself cannot induce leaf abscission, but it functions with ethephon to promote cotton defoliation to increase harvested crop quality (Burtom et al., 2008). CYC enhances the ethylene-induced efficiency of leaf abscission through possible inhibition of auxin transport or auxin signaling (Pedersen et al., 2006). CYC can also increase the ethephon defoliation efficiency at low temperatures. Ethephon cannot induce defoliation at a daily mean temperature below 28°C, but CYC combined with ethephon enhances defoliation at a daily mean temperature above 15°C (Pedersen et al., 2006).

TDZ combined with CYC can enhance defoliation at low temperatures (daily mean temperatures of 15°C, 17/13°C) (Liu et al., 2019), but the underlying mechanism remains unclear. Therefore, we compared the transcriptome and physiology in cotton AZs between the TDZ treatment and TDZ+CYC treatment at low temperatures to identify the reason why CYC can enhance TDZ defoliation efficiency at low temperatures. The results can help develop strategies promoting cotton defoliation at low temperatures.

## 2 Materials and methods

### 2.1 Plant materials

This research study was conducted in an artificial-climate laboratory at Jiangsu Academy of Agricultural Science, China. The cotton seedlings (Zhongmian 50) were grown according to our previous method (Shu et al., 2022). Cotton seedlings with five true leaves were treated with defoliants. Both sides of all leaves were evenly coated with defoliant using a brush. A total of three treatments were set up as follows: water (control, W), 0.1% TDZ application (TDZ, T), and a combined solution of 0.1% TDZ and 0.03% CYC (TDZ+CYC, TC). After drying the defoliant, these seedlings were placed at 15°C (day/night 17°C/13°C, low temperature, L). Then, the number of cotton leaves was recorded every day after treatment. For each treatment, the defoliation rate was calculated as the number of shed cotton leaves/total number of cotton leaves. The AZs (5 mm from petiole to distal end) of the fourth main cotton leaves were harvested at 24 and 144 h post-treatment, respectively. All AZ samples were stored at −70°C for transcriptomic sequencing and physiology analysis with three biological replicates for each treatment.

### 2.2 RNA extraction and sequencing

Total RNA was isolated from cotton leaf AZs. RNA quality was assessed using Anoroad (Beijing, China) prior to library construction. mRNA was purified from the total RNA. The samples described above were sequenced on an Illumina HiSeq platform, and 18 separate libraries were prepared. Sequencing data generated in this study were deposited in the NCBI SRA database (BioProject: PRJNA1061229). The sequenced genome data and annotation information of *G. hirsutum* (NAU-NBI_v1.1) were downloaded from the Cottongen (https://www.cottongen.org/) (Zhang et al., 2015). The mapped sequenced reads were normalized to the aligned expected number of fragments per kilobase of transcript sequence per million base pairs sequenced (FPKM) to obtain the relative expression levels of the identified genes (Trapnell et al., 2010).

Differentially expressed genes (DEGs) between these two groups were detected using the DEGseq package (Love et al., 2014). A *q*-value < 0.05 (Storey and Tibshirani, 2003) and |log_2_(Foldchange)| ≥ 1 was set as the threshold for significantly differential expressions. Gene ontology (GO) enrichment analysis of DEGs was evaluated using Blast2GO with a false discovery rate (FDR) < 0.05. Kyoto Encyclopedia of Genes and Genomes (KEGG) pathway enrichment of DEGs was performed using the KOBAS 2.0 software with an FDR < 0.05.

### 2.3 Determination of physiological indexes

Endogenous hormones were extracted according to a previous research study (Pan et al., 2010). The ACC, auxin, and JA contents were determined using high-performance liquid chromatography-tandem mass spectrometry.

The malondialdehyde (MDA) content was determined using the thiobarbituric acid method (Kucko et al., 2022b). The determination of H_2_O_2_ content was determined as described by Li et al. (2021).

### 2.4 Quantitative real-time PCR analysis

qRT-PCR was used to validate the RNA-seq results. The extraction of total RNA and the qRT-PCR reaction system and amplification process were the same as in our previous study (Shu et al., 2022). Relative expression levels were calculated using the 2^−ΔΔCT^ method (Livak and Schmittgen, 2001). The *actin* gene served as the internal reference. The qRT-PCR primers are listed in **Supplementary Table S1**.

### 2.5 Data analysis

Microsoft Excel 2016 was used for data organization. Statistical analyses were conducted using SPSS 17.0 (SPSS Institute Inc.). The treatment means were compared using Duncan’s multiple range tests at *P* < 0.05.

## 3 Results

### 3.1 CYC increased the TDZ defoliation rate at low temperatures

**Figure 1:**
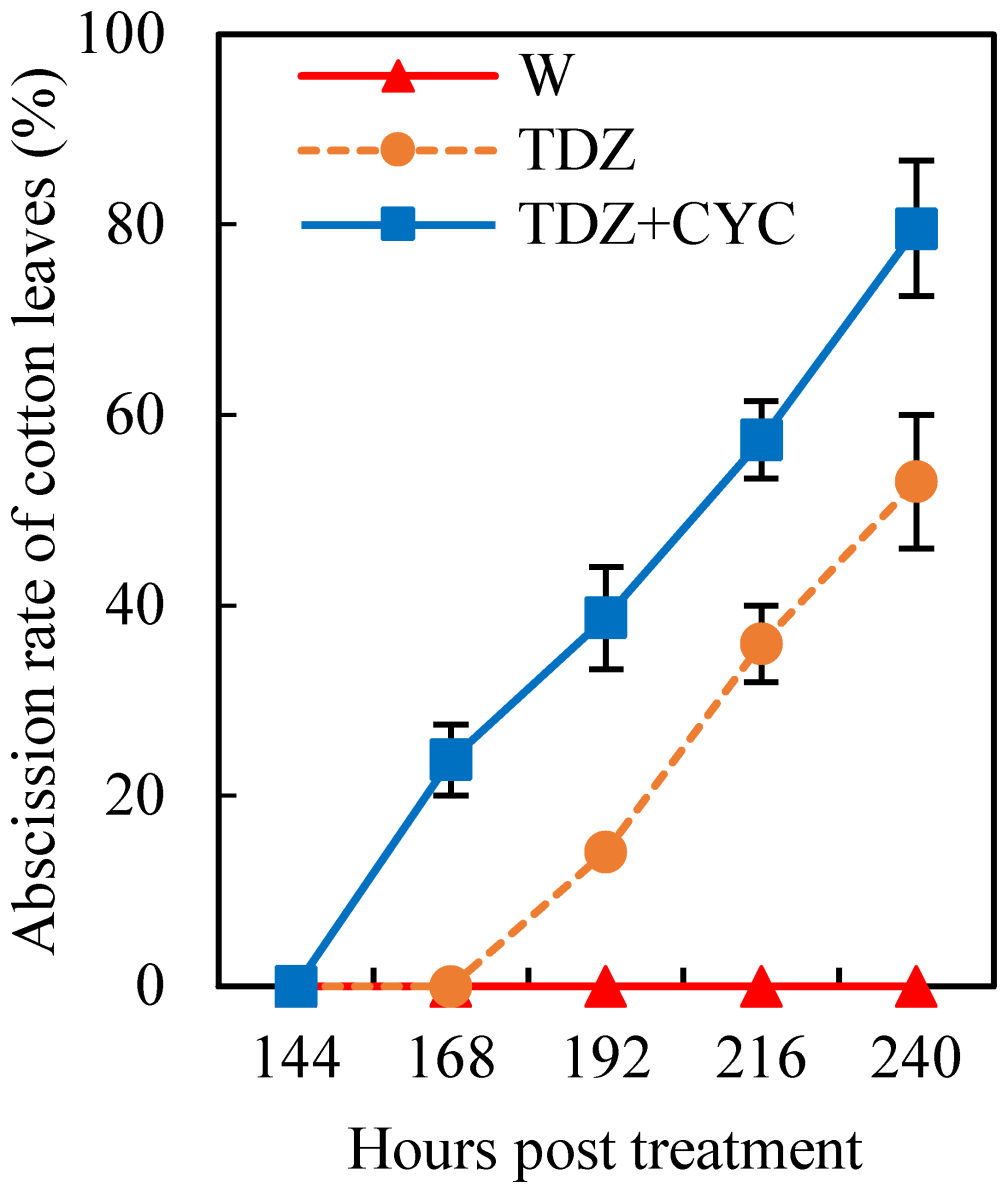
Cotton leaf abscission treated with defoliants at low temperatures. Defoliation rate (%) of cotton leaves treated with W, TDZ, and TDZ+CYC at 24~240 h post-treatment at 15°C. Error bars represent the SD of three biological replicates.

At low temperatures (daily mean temperature of 15°C), the leaves treated with TDZ began to abscise after 168 h, with a defoliation rate of ~53.0% at 240 h post-treatment. However, the start time of cotton leaf abscission after treatment with TDZ+CYC increased to 144 h post-treatment, and the defoliation rate increased to 79.6% at 240 h post-treatment (**Figure 1**).

### 3.2 Transcriptome changes in cotton AZs

We performed AZ transcriptome analysis at 24 and 144 h with W, T, and TC treatments, respectively. Clean reads of each sample exceeded 40 million. The Q30 values of clean data for all samples exceeded 91.27%, and all groups were able to map more than 90.14% of sequences to the genome (**Supplementary Table S2**). The results of the principal component analysis (PCA) and Pearson correlation analysis of all cDNA libraries (**Supplementary Figures S1A, B**) showed that the RNA-seq data were accurate and reliable and could be used for subsequent analysis.

**Figure 2:**
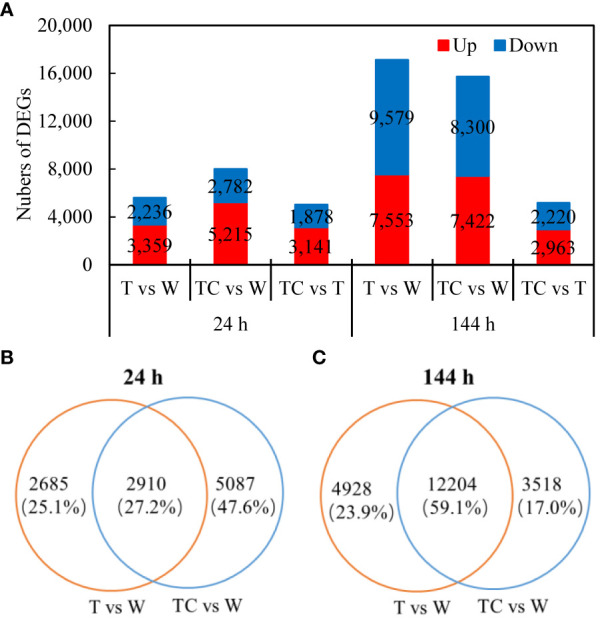
DEGs induced by defoliants in cotton AZs at low temperatures. **(A)**, the numbers of DEGs up- or downregulated at 24 and 144 h post-treatment. The DEGs are controlled by *q*-value < 0.05 and |log_2_FC| ≥ 1. FC represents the expression-level fold change in genes. **(B, C)**, Venn diagram displaying the numbers of DEGs unique or overlapping between T vs. W and TC vs. W at 24 h post-treatment **(B)** or 144 h post-treatment **(C)**. Black digits indicate the numbers of DEGs. The percentage shows the proportion of each type of DEGs among all DEGs.

Compared with the W treatment at 24 h, 5595 DEGs (3359 up- and 2236 downregulated genes) and 7997 DEGs (5215 up- and 2782 downregulated genes) in AZs were identified for the TDZ and TDZ+CYC treatments, respectively (**Figure 2A**). At 144 h post-treatment, 17132 DEGs (7553 up- and 9579 downregulated genes) and 15722 DEGs (7422 up- and 8300 downregulated genes) were identified for the TDZ and TDZ+CYC treatments, respectively (**Figure 2A**). These results indicated that the number of DEGs increased with prolonged defoliant treatment. Compared with the TDZ treatment alone, TDZ+CYC induced more DEGs at 24 h post-treatment at low temperatures.

A Venn diagram was used to display the numbers of DEGs induced by the TDZ and TDZ+CYC treatments. At 24 h post-treatment, among the 5595 DEGs induced by TDZ, 2910 DEGs were also induced by the TDZ+CYC treatment. A total of 5087 DEGs were specially induced by the TDZ+CYC treatment (**Figure 2B**). At 144 h post-treatment, among the 17132 DEGs induced by TDZ, 12204 DEGs were also induced by TDZ+CYC. A total of 3518 DEGs were specially induced by TDZ+CYC (**Figure 2C**). Compared with 24 h post-treatment, the proportion of DEGs regulated by both TDZ and TDZ+CYC significantly increased at 144 h. Approximately 64% (5087 unique DEGs/7997 DEGs) of DEGs induced by TDZ+CYC differed from those induced by TDZ at 24 h post-treatment. At 144 h post-treatment, the difference between these two treatments decreased.

**Figure 3:**
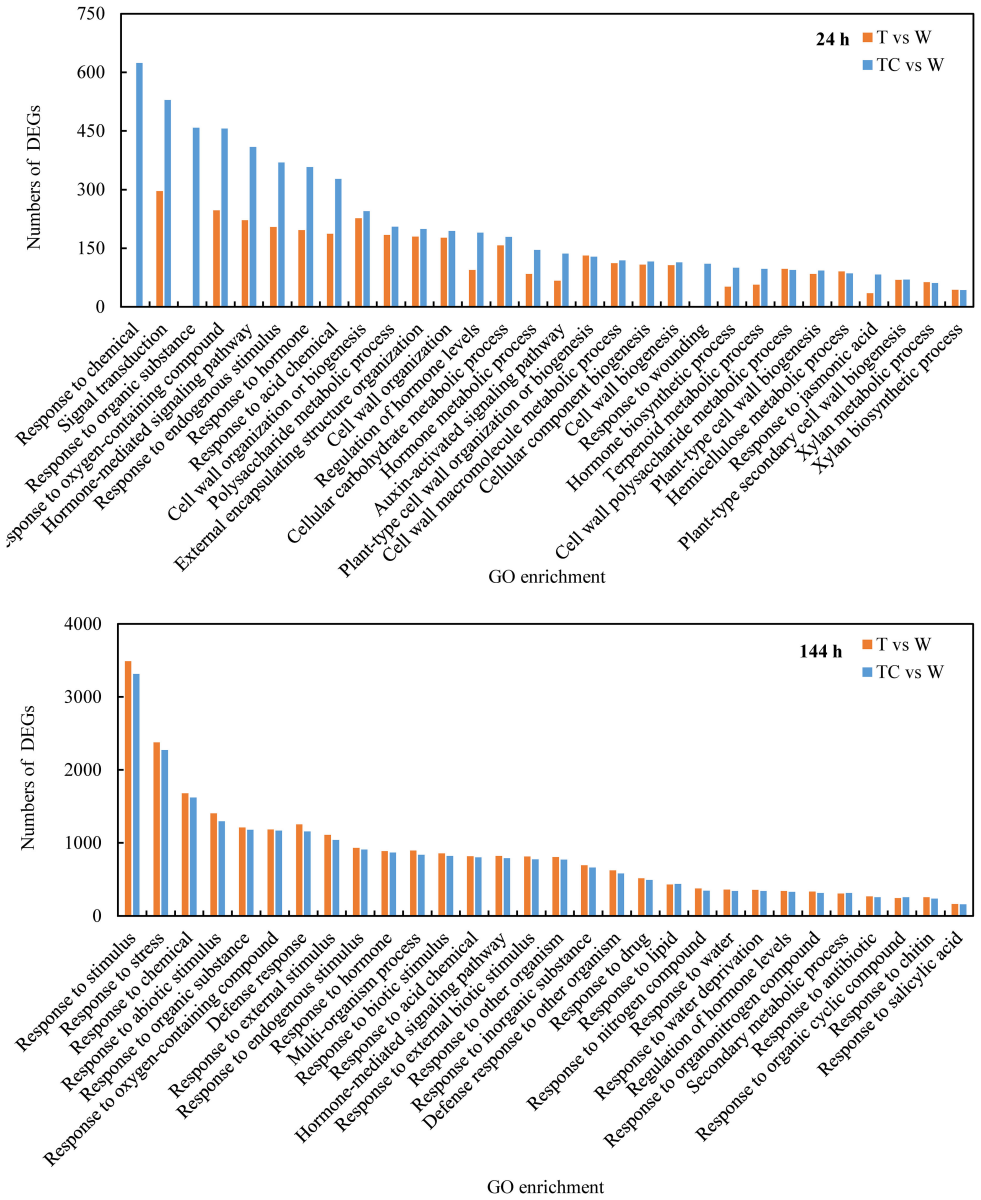
Top 30 GO terms (FDR < 5*10^-13^) of DEGs in biological process at 24 h and 144 h post-treatment at low temperatures. The Y-axis shows the numbers of DEGs, and the X-axis shows the enriched GO enrichments of DEGs. T vs. W represents DEGs induced by the TDZ treatment compared with the control. TC vs. W represents DEGs induced by the TDZ+CYC treatment compared with the control.

A GO enrichment analysis was performed to classify the function of the identified DEGs that were induced by TDZ or TDZ+CYC. The top 30 GO terms in the biological process category of TDZ vs. control and TDZ+CYC vs. control (FDR<5×10^-13^) are shown in **Figure 3**. At 24 h post-treatment, the gene numbers of half of the 30 GO items differed significantly between these two treatments. Among them, eight GO items (“signal transduction,” “hormone-mediated signaling pathway,” “response to hormones,” “regulation of hormone levels,” “hormone metabolic process,” “auxin-activated signaling pathway,” “hormone biosynthetic process,” and “response to jasmonic acid”) were related to hormones, and one GO item “response to oxygen-containing compounds” was related to ROS pathways. At 144 h post-treatment, the enriched GO items for these two treatments were similar. These results suggested that compared with the TDZ treatment alone, TDZ+CYC could induce the expression of some genes related to plant hormone biosynthetic, metabolic, and signal transduction processes and the ROS pathway at an early stage.

**Figure 4:**
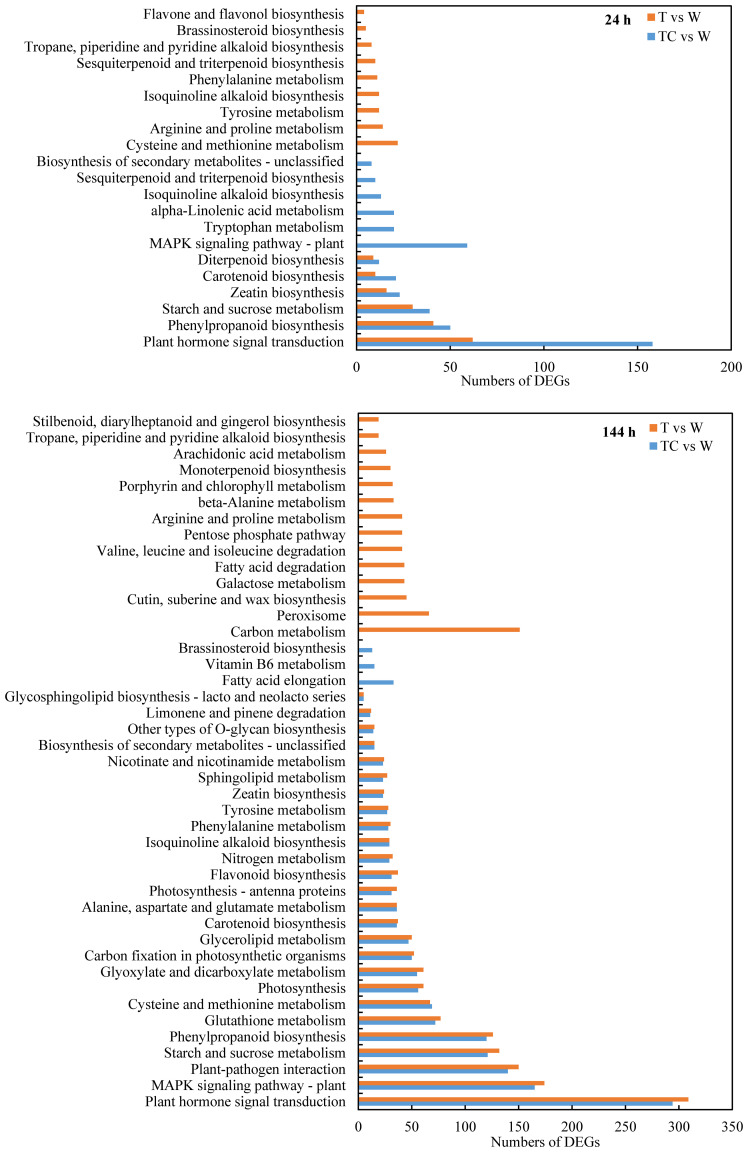
Enriched KEGG pathways (FDR < 0.05) of DEGs at 24 h and 144 h post-treatment at low temperatures. The X-axis shows the numbers of DEGs, and the Y-axis shows the enriched KEGG pathways of DEGs. T vs. W represents DEGs induced by the TDZ treatment compared with the control. TC vs. W represents DEGs induced by the TDZ+CYC treatment compared with the control.

KEGG pathway enrichment analysis was performed to identify the DEG functions (**Figure 4**). At 24 h post-treatment, six pathways (“plant hormone signal transduction,” “phenylpropanoid biosynthesis,” “starch and sucrose metabolism,” “zeatin biosynthesis,” “carotenoid biosynthesis,” and “diterpenoid biosynthesis”) were enriched in both the TDZ vs. control and TDZ+CYC vs. control comparisons. At 144 h post-treatment, among the 29 KEGG pathways regulated by TDZ+CYC, 26 KEGG pathways were also regulated by TDZ. The top six KEGG pathways were “plant hormone signal transduction,” “MAPK signaling pathway-plant,” “plant-pathogen interactions,” “starch and sucrose metabolism,” “phenylpropanoid biosynthesis,” and “glutathione metabolism.” The “plant hormone signal transduction” pathway might be an important KEGG term, which was induced by TDZ or TDZ+CYC at 24 and 144 h post-treatment. The numbers of DEGs in this pathway differed significantly between these two treatments at 24 h. TDZ only induced 62 DEGs, while TDZ+CYC induced 158 DEGs. However, there was no significant difference between these two treatments at 144 h post-treatment. These results indicated that TDZ+CYC induced the expression of plant hormone response related genes in cotton AZs at the early stage at low temperatures.

### 3.3 Plant hormone pathways in response to defoliants

**Figure 5:**
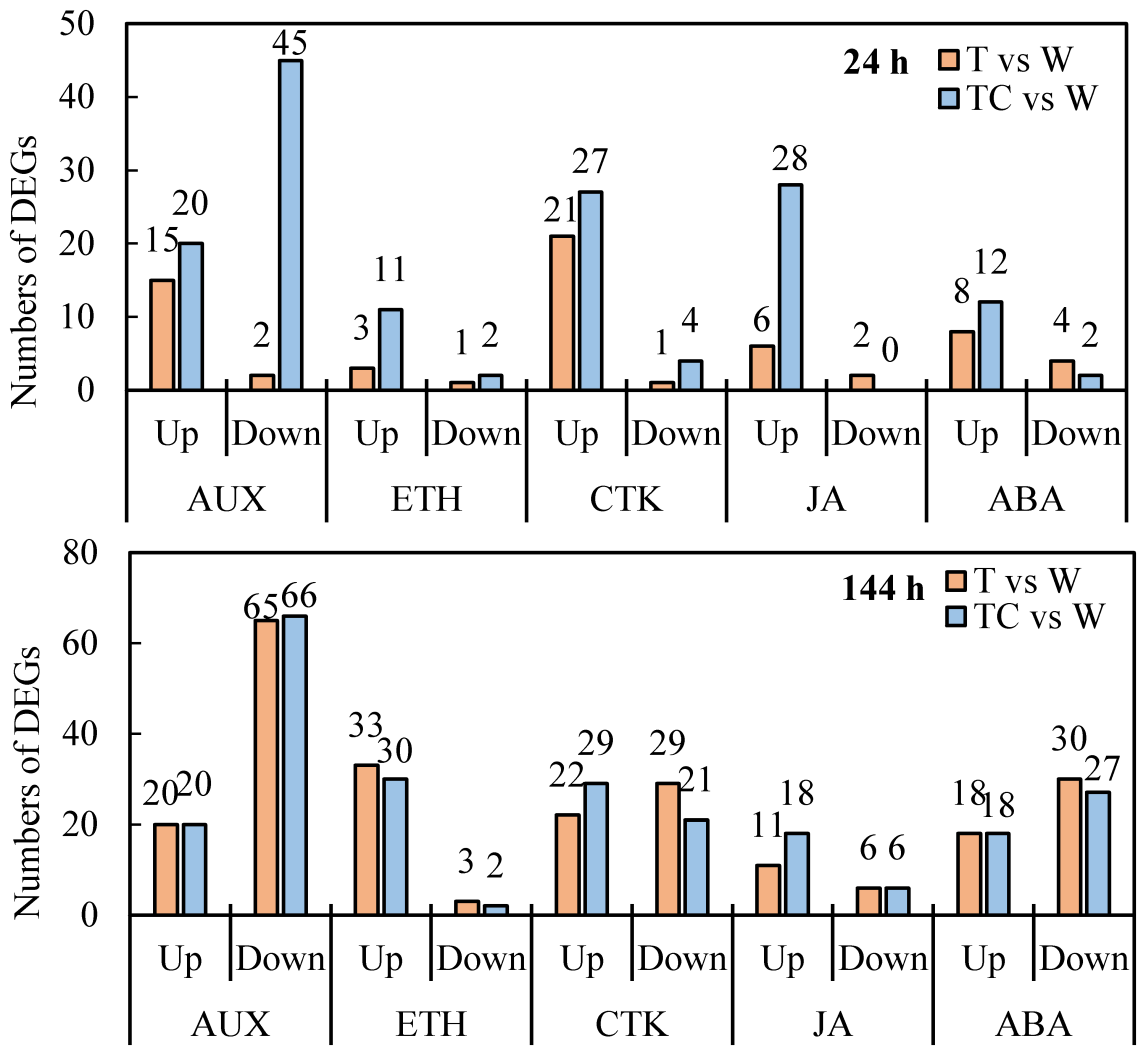
The numbers of DEGs related to plant hormone response pathways in cotton AZs treated by defoliants at low temperatures. AUX, Auxin. ETH, ethylene. CTK, cytokinin. JA, jasmonic acid. ABA, abscisic acid. Up represents upregulated DEGs. Down represents downregulated DEGs. T vs. W represents DEGs induced by the TDZ treatment compared with the control. TC vs. W represents DEGs induced by the TDZ+CYC treatment compared with the control. Black digits indicate the numbers of DEGs.

Next, auxin, ethylene, cytokinin, JA, and abscisic acid (ABA) response related genes, including those that encode for transport, signaling and response factors, induced by TDZ or TDZ+CYC were analyzed. At 24 h post-treatment, TDZ and TDZ+CYC upregulated the expressions of 15 and 20 auxin response related genes, 3 and 11 ethylene response related genes, and 6 and 28 JA response related genes in cotton AZs, respectively (**Figure 5**). TDZ and TDZ+CYC downregulated the expression of 2 and 45 auxin response related genes, 1 and 2 ethylene response related genes, and 2 and 0 JA response related genes in cotton AZs, respectively (**Figure 5**). These results showed that the numbers of downregulated auxin response related genes, upregulated ethylene response related genes, and upregulated JA response related genes between these two treatments differed at 24 h post-treatment.

**Figure 6:**
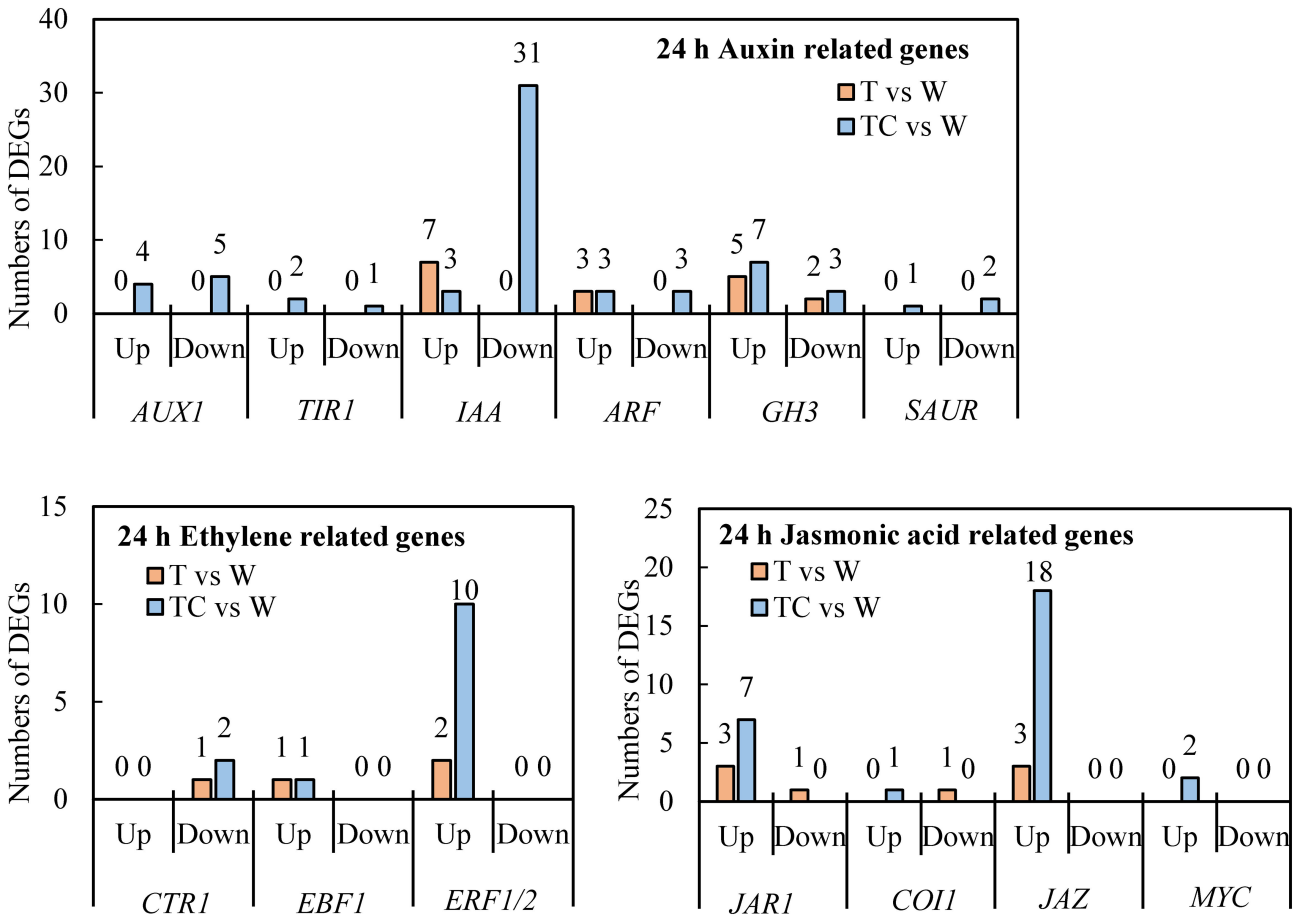
The numbers of DEGs related to auxin, ethylene, and JA response pathways in cotton AZs treated with defoliants at low temperatures. The DEGs are controlled by q-value < 0.05 and |log_2_FC| ≥ 1. Up represents upregulated DEGs. Down represents downregulated DEGs. T vs. W represents DEGs induced by the TDZ treatment compared with the control. TC vs. W represents DEGs induced by the TDZ+CYC treatment compared with the control. Black digits indicate the numbers of DEGs.

**Figure 7:**
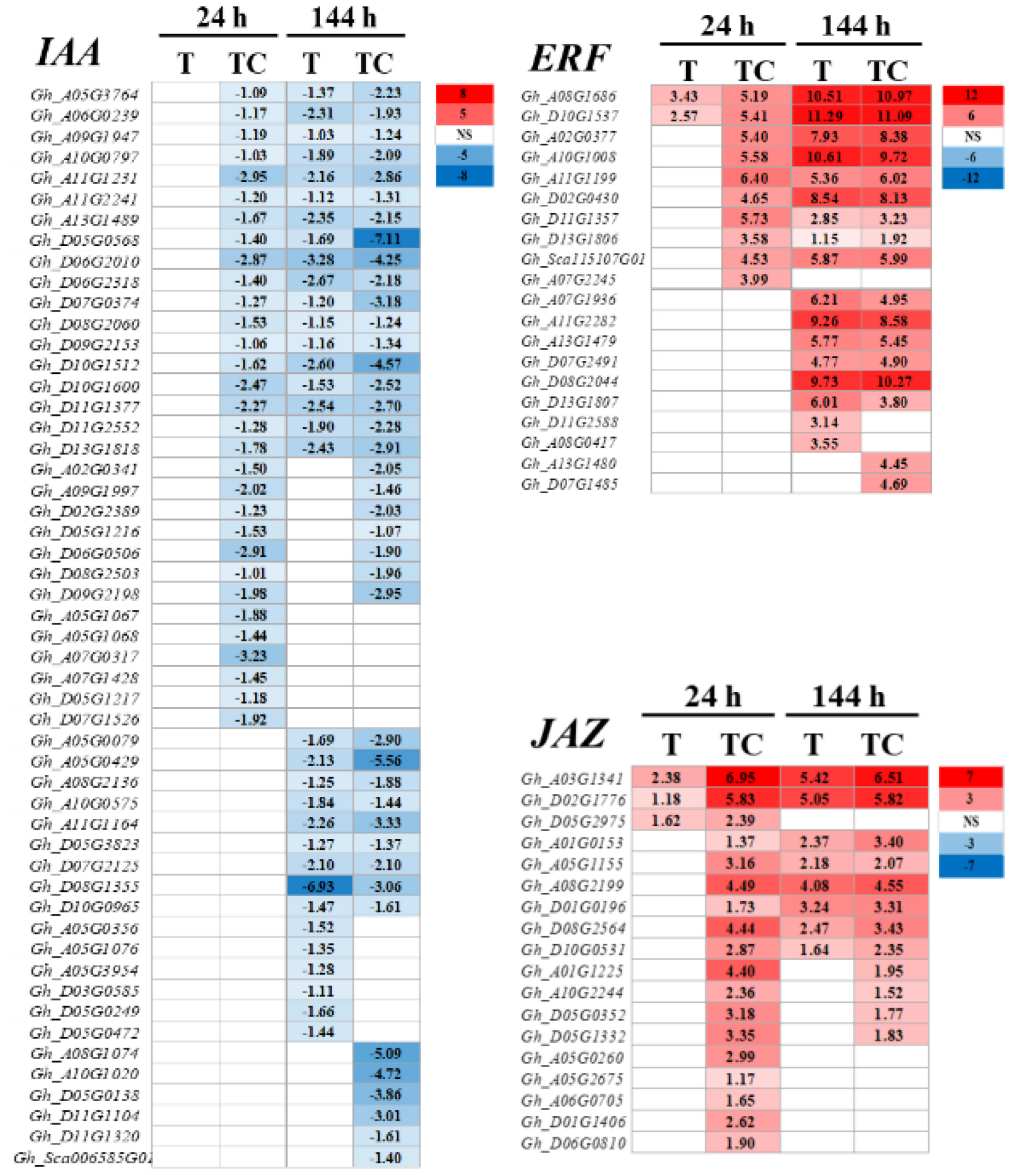
Heatmap of downregulated *IAA* genes and upregulated *ERF* and *JAZ* genes generated based on RNA-seq data. The number in the box indicates the value of Log_2_FC (the values of Log_2_FC ≥1 are shown). FC represents the expression-level fold change in genes. “NS” represents |log_2_FC| < 1.

TDZ+CYC could downregulate more auxin response related genes, including *AUX1* (*AUXIN TRANSPORTER PROTEIN 1*), *TIR1* (*TRANSPORT-INHIBITOR RESPONSE 1*), *IAAs* (*INDOLE-3-ACETIC ACID*), *ARFs* (*AUXIN-RESPONSE FACTORS*), *GH3* (*GRETCHEN HAGEN 3*, GH3s conjugated amino acids to IAA) and *SAURs* (*SMALL-AUXIN UPREGULATED RNAs*), compared with TDZ at 24 h post-treatment (**Figure 6**). *IAA* genes showed the greatest difference between these two treatments. A total of 31 *IAA* genes were downregulated in AZs treated with TDZ+CYC, while no *IAA* genes were regulated by TDZ alone. At 144 h post-treatment, more *IAA* genes were downregulated, but the difference between these two treatments decreased. Totals of 33 and 40 *IAA* genes were downregulated in TDZ and TDZ+CYC, respectively (**Figure 7** and **Supplementary Figure 2S**). In addition, the expression levels of most *IAA* genes induced by TDZ+CYC were lower than those induced by TDZ. Compared with the TDZ treatment alone, TDZ+CYC could downregulate the expression of auxin response related genes at the early stage at low temperatures.

Ethylene response related genes, including *CTR1* (*CONSTITUTIVE TRIPLE RESPONSE 1*), *EBF1* (*EIN3-BINDING F-BOX*), and *ERF1* (*ETHYLENE-RESPONSE FACTORS*) genes, were induced by TDZ and TDZ+CYC (**Figure 6** and **Supplementary Figure 2S**). The numbers of upregulated *ERF1/2* genes differed between these two treatments. TDZ+CYC and TDZ upregulated the expression of 10 and 2 *ERF1/2* genes in AZs, respectively. At 144 h post-treatment, the difference in *ERF1/2* gene numbers between these two treatments decreased. Both treatments upregulated the expression of 17 *ERF1/2* genes in AZs (**Figure 7**). These results indicated that TDZ+CYC could upregulate the expression of *ERF1/2* genes in AZs at the early stage at low temperatures.

JA response related genes, including *JAR1* (*JASMONATE RESISTANT* 1), *COI-1* (*CORNATINE INSENSITIVE 1*), *JAZ* (J*SMONATE ZIM-DOMAIN*), and *MYC* genes, were induced by TDZ and TDZ+CYC (**Figure 6** and **Supplementary Figure 2S**). The numbers of all identified upregulated JA response related genes induced by TDZ+CYC exceeded those induced by TDZ, especially for the *JAZ* gene. TDZ and TDZ+CYC upregulated 3 and 18 *JAZ* genes, respectively. TDZ induced more upregulated *JAZ* genes at 144 h than at 24 h, while TDZ+CYC showed an inverse effect. At 144 h post-treatment, TDZ and TDZ+CYC upregulated the expression of 8 and 12 *JAZ* genes, respectively (**Figure 7**). These results indicated that TDZ+CYC could induce the expression of JA response related genes (*JAR1*, *COI-1*, *JAZ*, and *MYC*) at the early stage at low temperatures.

**Figure 8:**
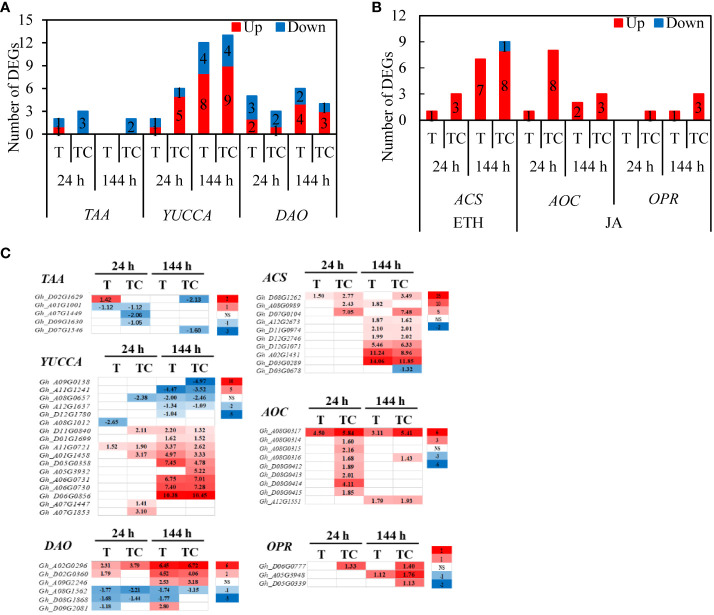
The expression of DEGs in plant hormone synthesis and metabolism pathway. **(A)** Numbers of DEGs in auxin biosynthesis and metabolism pathways in AZs treated with defoliants at low temperatures. **(B)** Numbers of DEGs in ETH and JA biosynthesis and metabolism pathways in AZs treated with defoliants at low temperatures. The DEGs are controlled by q-value < 0.05 and |log_2_FC|≥ 1. ETH, ethylene. JA, jasmonic acid. Up represents upregulated DEGs. Down represents downregulated DEGs. T represents DEGs induced by the TDZ treatment compared with the control. TC represents DEGs induced by the TDZ+CYC treatment compared with the control. Black digits indicate the numbers of DEGs. **(C)** Heatmap of *TAA*, *YUCCA*, *DAO*, *ACS*, *AOC*, and *OPR* genes generated based on RNA-seq data. The number in the box indicates the value of Log_2_FC (the values of Log_2_FC ≥1 are shown). FC represents the expression-level fold change in genes. “NS” represents |log_2_FC| < 1.

Some genes in the plant hormone biosynthesis and metabolic pathway had different responses to TDZ or TDZ+CYC (**Figures 8A, B**). TDZ+CYC downregulated the expression of 3 and 2 *TAA* (*TRYPTOPHAN AMINOTRANSFERASE*, auxin synthesis gene) genes, while only 1 and 0 *TAA* genes were downregulated by TDZ at 24 h and 144 h post-treatment, respectively (**Figure 8A**). TDZ+CYC upregulated the expression of 3 *ACS* (*1-AMINOCYCLOPROPANE-1-CARBOXYLATE SYNTHASEs*, ethylene synthesis gene) genes, 8 *AOC* (*ALLENE OXIDE CYCLASEs*, JA synthesis gene) genes, and 1 *OPR* (*12-OXO-PHYTODIENOATE REDUCTASE*, JA synthesis gene) gene. In contrast, only 1 *ACS* gene and 1 *AOC* gene were upregulated by TDZ at 24 h post-treatment (**Figures 8B, C**).

**Figure 9:**
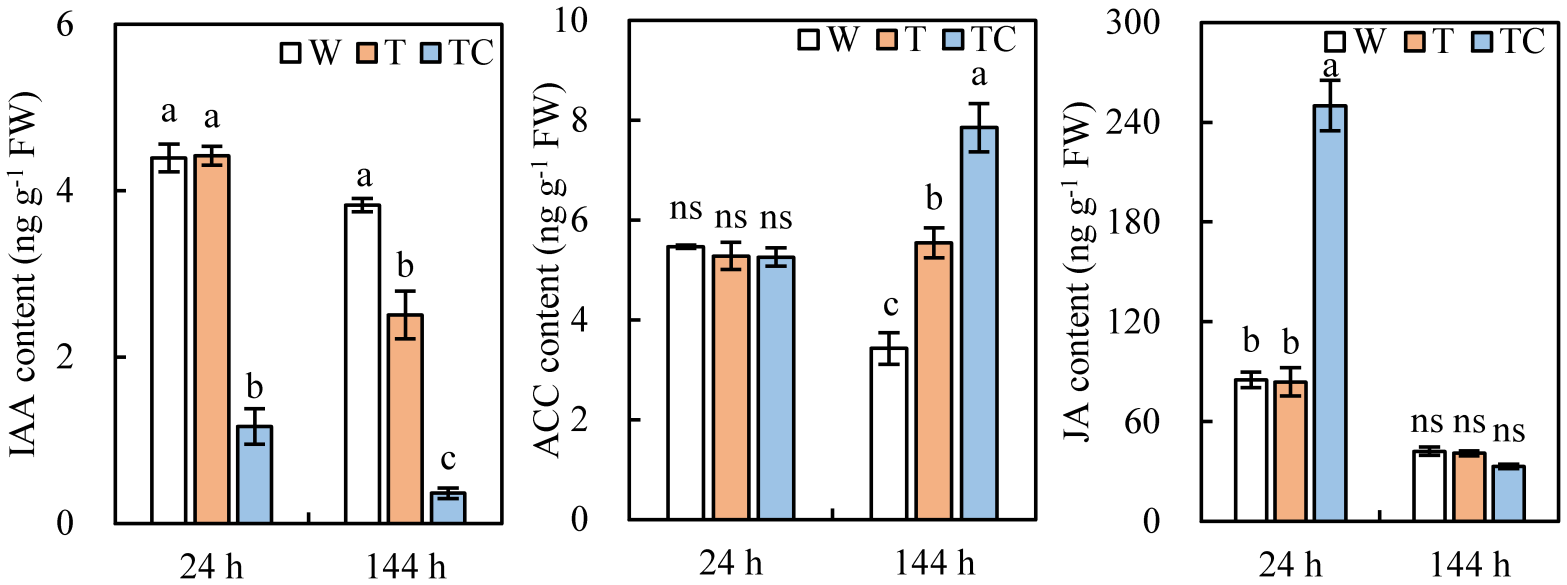
The auxin, ethylene, and JA contents in AZs treated with defoliants at low temperatures. Different lowercase letters at the same time point indicate significant differences at the 0.05 level, ns indicates no significant difference at the 0.05 level. W represents the control treatment. T represents the TDZ treatment. TC represents the TDZ+CYC treatment.

Compared with the W treatment, the IAA content in TDZ-treated AZs decreased at 144 h post-treatment at low temperatures. It occurred at 24 h post-treatment in AZs treated with TDZ+CYC (**Figure 9**). Both TDZ and TDZ+CYC induced an increase in the ACC content at 144 h post-treatment, while the ACC content with the TDZ+CYC treatment was higher than the TDZ treatment alone (**Figure 9**). The TDZ treatment alone failed to change the JA content in AZs. TDZ+CYC induced a significant increase in JA content in AZs at 24 h post-treatment (**Figure 9**). Compared with TDZ, the application of TDZ+CYC could decrease the IAA content and increase the JA and ACC contents in cotton AZs at low temperatures.

### 3.4 ROS pathway in response to defoliants

**Figure 10:**
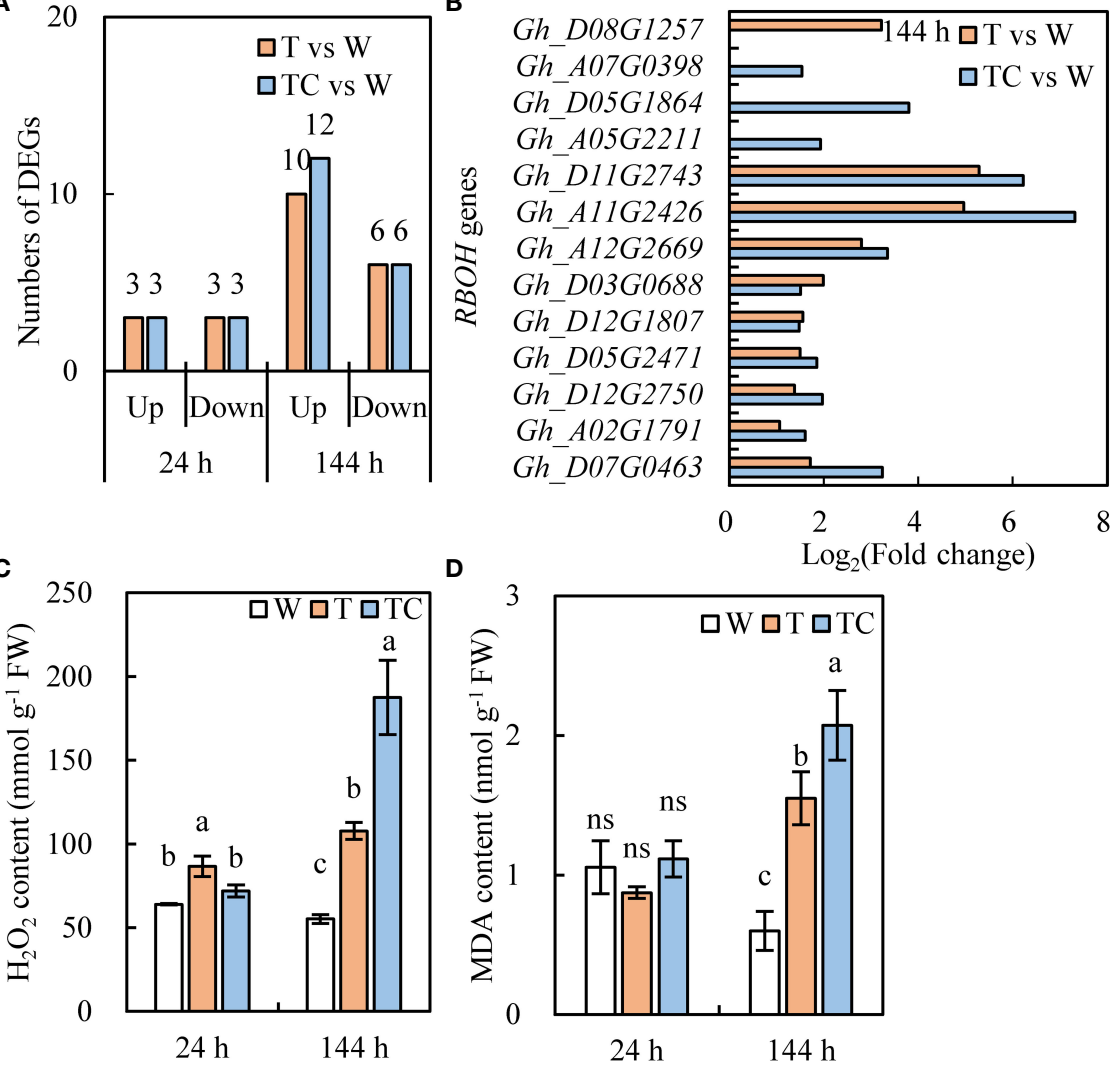
DEGs and physiology analysis of ROS pathway in AZs treated with defoliants at low temperatures. **(A)** the number of *RBOH* genes. Black digits indicate the numbers of DEGs. **(B)** the expression level of *RBOH* genes in AZs at 144 h. T vs. W represents, compared with the control, the DEGs induced by the TDZ treatment. TC vs. W represents, compared with the control, the DEGs induced by the TDZ+CYC treatment. **(C)** the content of H_2_O_2_. **(D)** the content of MDA. W represents the control treatment. T represents TDZ treatment. TC represents TDZ+CYC treatment. Different lowercase letters in the same time point indicate significant differences at the 0.05 level, ns indicates no significant difference at the 0.05 level.

ROS plays a critical role in the organ-shedding process caused by TDZ (Jin et al., 2020). The numbers of *RBOH* genes induced by the TDZ and TDZ+CYC treatments were similar (**Figure 10A**). However, among the upregulated *RBOH* genes, the expression levels of most genes in the TDZ+CYC treatment were higher than in the TDZ treatment at 144 h post-treatment (**Figure 10B**).

TDZ+CYC induced a higher H_2_O_2_ content in AZs at 144 h post-treatment than TDZ (**Figure 10C**). Compared with the W treatment, TDZ and TDZ+CYC induced a significant increase in the MDA content in AZs at 144 h post-treatment. Moreover, the MDA content in AZs with TDZ+CYC treatment was higher than that of the TDZ treatment (**Figure 10D**). These results showed that compared with TDZ alone, TDZ+CYC enhanced ROS production in AZs at low temperatures.

### 3.5 Cell wall hydrolase genes in response to defoliants

**Figure 11:**
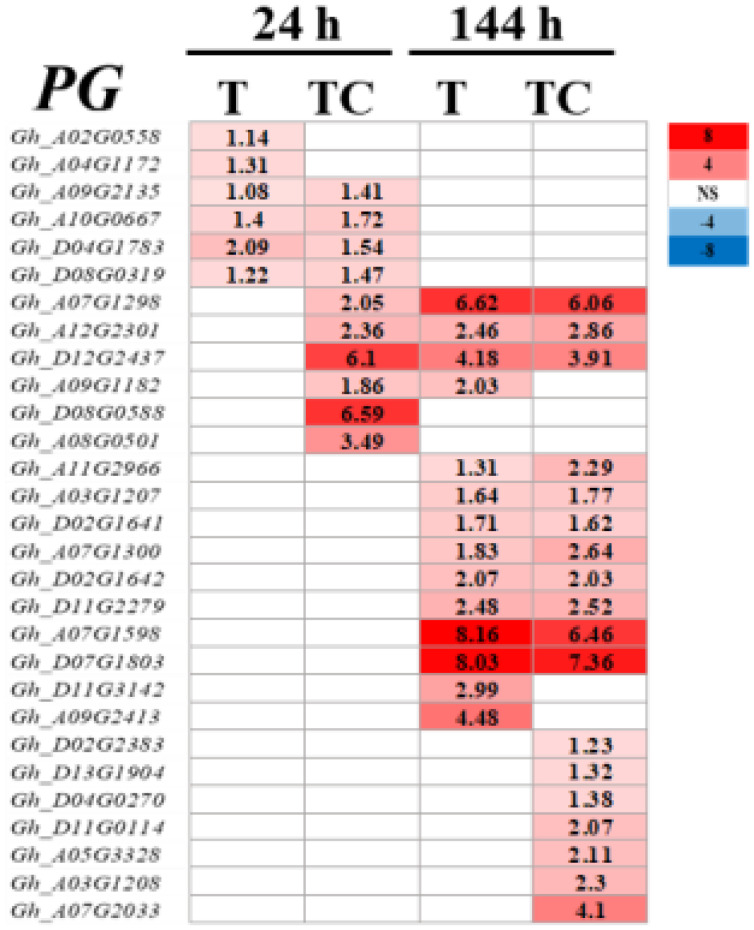
Heatmap of *PG* genes generated based on RNA-seq data. The number in the box indicates the value of Log_2_FC (the value of Log_2_FC ≥1 is shown). FC represents the expression-level fold change in genes. “NS” represents |log_2_FC| < 1.

Polygalacturonase (PG) is a major cell wall hydrolase (Gonzalez-Carranza et al., 2002). *PG* genes were induced by TDZ and TDZ+CYC at low temperatures (**Figure 11**). Compared with TDZ alone, more *PG* genes were upregulated in AZs treated with TDZ+CYC at 24 h and 144 h post-treatment at low temperatures.

### 3.6 Transcription factors in response to defoliants

**Figure 12:**
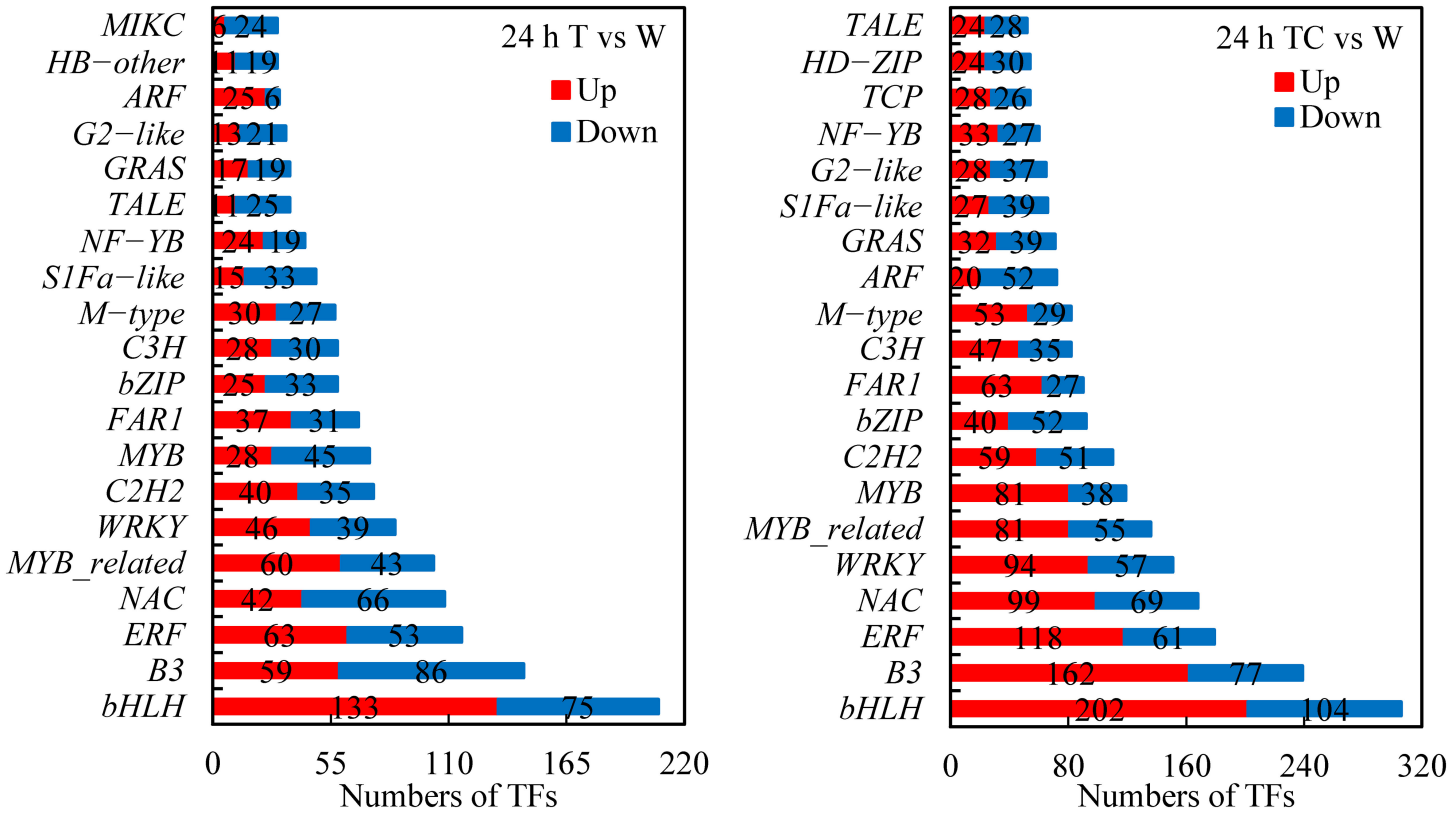
Numbers of TFs in AZs treated with defoliants at low temperatures. T vs. W represents TFs induced by the TDZ treatment compared with the control. TC vs. W represents TFs induced by the TDZ+CYC treatment compared with the control. Up represents upregulated TFs. Down represents downregulated TFs. Black digits indicate the numbers of differentially expressed TFs.

TFs play important roles in regulating gene expression related to organ shedding (Huang et al., 2019; Zhang et al., 2019; Zhao et al., 2020). In this study, 1849 and 2842 differentially expressed TFs were found in cotton AZs treated with TDZ or TDZ+CYC, respectively. These TFs included bHLH, B3, ERF, MYB, WRKY, NAC, etc. (**Figure 12**). TDZ+CYC activated more TFs than TDZ alone. Among the top 20 TFs, 18 were regulated by both treatments. Moreover, the numbers of each kind of TF in the TDZ+CYC group exceeded the TDZ group. The downregulated ARF number in the TDZ treatment is only 6, while that of TDZ+CYC is up to 52. Compared with the TDZ treatment, the numbers of upregulated bHLH, B3, ERF, NAC, and MYB TFs in the TDZ+CYC treatment were increased by over 50. The results indicated that ARF, bHLH, B3, ERF, NAC, and MYB might be involved in early signal transduction in TDZ+CYC at low temperatures.

### 3.7 Validation of RNA-seq results

qRT-PCR was used to confirm the gene expression levels obtained using RNA-sequencing. Five genes were randomly selected from different pathways responsive to TDZ+CYC. The results showed that expression data from qRT-PCR were in accordance with RNA-seq data (**Supplementary Figure S3**), which confirmed the reliability of RNA-seq analyses in this study.

## 4 Discussion

Our previous research (Shu et al., 2022) showed that at normal temperatures (daily mean temperature of 25°C), all leaves treated with TDZ dropped at 96 h post-treatment. However, low temperatures (daily mean temperature of 15°C) inhibited cotton leaf abscission induced by TDZ (Shu et al., 2022), and the defoliation rate in TDZ at low temperatures cannot meet the demand for mechanized cotton harvesting. In this study, compared with the TDZ treatment alone, TDZ+CYC was able to accelerate defoliation and increase the cotton leaf defoliation rate by approximately 80% at low temperatures. CYC alone does not induce defoliation (Elfving and Visser, 2005), but it could be used as a synergist to enhance ethephon activity (Pedersen et al., 2006). The result of this study showed that TDZ combined with CYC significantly enhanced cotton leaf abscission at low temperatures.

### 4.1 CYC enhanced the TDZ defoliation efficiency by regulating plant hormones

In the plant organ abscission process, abscission signaling and enzymatic hydrolysis of the middle lamella are two key phases regulated by TDZ in cotton AZs (Li et al., 2022). TDZ regulates the plant hormone synthesis and signaling transduction in AZs to induce leaf abscission (Du et al., 2014; Xu et al., 2019; Jin et al., 2020), while the role of TDZ is delayed and reduced at low temperatures (Shu et al., 2022). In this study, compared with the TDZ treatment alone, a combination of TDZ and CYC could activate plant hormone synthesis and response related genes earlier at low temperatures. The plant hormones auxin and ethylene are the main plant growth regulators that control natural leaf abscission (Li et al., 2022). While auxin suppresses abscission, ethylene promotes it (Taylor and Whitelaw, 2001). In this study, compared with the TDZ treatment, TDZ+CYC not only affected auxin and ethylene synthesis and response related pathways but also regulated the JA synthesis and response related pathways.

Auxin functions as a brake to regulate abscission, and a high auxin content in AZs inactivates organ abscission (Basu et al., 2013; Xie et al., 2015; Kucko et al., 2020). Low temperatures inhibit the response of most auxin synthesis and signaling genes to TDZ, which results in the auxin content in AZs not decreasing and an auxin gradient not being formed (Shu et al., 2022). CYC is an auxin transport inhibitor (Pedersen et al., 2006). This study found that compared with the TDZ treatment alone, TDZ combined with CYC downregulated the auxin biosynthesis (*TAA*) and response related genes (*ARF*, *AUX1*, and *IAA*) and decreased the auxin content at the early stage at low temperatures. *TAA* is required for auxin production (Sun et al., 2016). The downregulated *TAA* affected the synthesis of auxin. *GH3* genes affected the free IAA content (Staswick et al., 2005), but the numbers of downregulated and upregulated *GH3* were both increased in TDZ+CYC treatment. The change of *GH3* might not the key factor for the decrease of auxin content in TDZ+CYC treatment. *IAA* genes were significantly regulated by TDZ+CYC at low temperatures. Previous study showed that downregulation of *RhIAA16* promoted rose petal abscission (Gao et al., 2016). These indicated that *IAA* genes play important role in the regulation of leaf abscission by TDZ+CYC at low temperatures. Therefore, the decreased auxin content and downregulated auxin response related genes in AZs treated with TDZ+CYC at low temperatures were beneficial for forming an auxin gradient and a hormonal imbalance in cotton leaf AZs, which could increase the effect of ethylene (Pedersen et al., 2006) and enhance abscission.

Ethylene is the main hormone that promotes plant organ abscission by inducing the cell wall hydrolase production in AZs (Mishra et al., 2008). At low-temperature conditions, most ethylene synthesis and response related genes slowly responded to TDZ, which resulted in the ethylene content in cotton AZs not rapidly increasing and a failure to activate downstream gene expression (Shu et al., 2022). In this study, TDZ+CYC upregulated *ERF* genes and enhanced the ethylene content in AZs. In *Rosa hybrida*, *ERF1* and *ERF4* mediated petal abscission by influencing pectin degradation in AZs (Gao et al., 2019). Therefore, the increased ethylene content and the upregulated ethylene response related genes (*ERF*) in AZs treated with TDZ+CYC at low temperatures could enhance cotton leaf abscission.

JA has also been found to be involved in plant organ shedding, and in this process, it interacts with auxin and ethylene (Saniewski et al., 2000). The JA-Me-induced formation of secondary AZs in the stems of *Bryophyllum calycinum* was inhibited by auxin (Saniewski et al., 2000). Studies on leaf and fruit abscission in various plants found that JA accumulation in AZs might induce abscission by promoting ethylene synthesis (Ueda et al., 1996; Fidelibus et al., 2022; Liu et al., 2022; Kucko et al., 2022a). This study found that at low temperatures, compared with TDZ, TDZ+CYC mainly induced the upregulation of JA synthesis gene (*AOC4 and OPR*) and response related genes (*JAR1*, *COI1*, *MYC*, and *JAZ*) in AZs during the early stage of abscission, increasing the JA content in AZs. The mutants of opr3 or coi1 have a delayed flower abscission phenotype (Park et al., 2002; Zhang et al., 2020). A previous research study found that after the AZ auxin polar flow was impaired, the expression of *SlJAR1* increased, leading to JA-Ile accumulation (Liu et al., 2022). JA upregulated cellulase activity in the leaf AZs of *P. vulgaris*, which led to cell wall polysaccharide degradation (Ueda et al., 1996). These indicated that at low temperatures, TDZ combined with CYC induced the upregulation of *JAR*, *COI1*, and *MYC*, which was beneficial for degrading cell wall polysaccharides in AZs and promoting cotton leaf abscission. However, more upregulated *JAZ* genes might inhibit JA signaling because the transcriptional inhibitor jasmonate ZIM domain (JAZ) protein binds the transcription factor MYC2 to repress JA signaling. The transcriptional repressor protein OsJAZ1 inhibits JAs in drought-treated rice (Fu et al., 2017; Wang et al., 2020). Once JA-Ile accumulates in the cytosol, it is transported to the nucleus (Li et al., 2017) and binds to COI1, which triggers degradation of the repressors—JAZ (Thines et al., 2007; Kucko et al., 2022a). This might be the reason why the JA content and the numbers of upregulated *JAZ* genes decreased in cotton leaf AZs treated by TDZ+CYC for 144 h at low temperatures.

### 4.2 CYC enhanced the TDZ defoliation efficiency by regulating the ROS system

ROS is a signaling substance that can regulate many plant physiological processes (Kim, 2014; Liao et al., 2016b). ROS plays an important role in the abscission process, and related genes are obviously induced by TDZ in AZs (Sakamoto et al., 2008b; Li et al., 2021). TDZ significantly increased the expression of the *RBOH* gene and the H_2_O_2_ content when inducing cotton leaf abscission (Li et al., 2021). In this study, compared with the TDZ treatment alone, TDZ combined with CYC increased the expression level of most of the upregulated *RBOH* genes at 144 h and significantly promoted the accumulation of H_2_O_2_ and MDA in the AZs at low temperatures. H_2_O_2_ is responsible for maintaining the proper ROS balance in AZs (Kucko et al., 2022b), and it regulates cell wall-degrading enzyme gene expression (Sakamoto et al., 2008a) and loosens the cell wall in the abscission process. In this study, cell wall hydrolase (PG) genes in AZs were activated by TDZ combined with CYC at the early stage at low temperatures. Therefore, an increase in ROS in AZs treated by TDZ and CYC might act as an abscission accelerator at low temperatures.

### 4.3 TDZ combined with CYC induced changes in TFs

TFs play key roles in plant development, and some TFs were involved in plant organ abscission. TDZ could regulate the expression of a large number of TFs, including Zinc finger, MYB, bHLH, ERF, WRKY, NAC, Homeobox, etc (Li et al., 2022). In this study, compared with the TDZ treatment alone, TDZ combined with CYC upregulated more bHLH, B3, ERF, NAC, and MYB TFs and downregulated more ARF TFs in AZs at low temperatures. The bHLH TF family is associated with the abscission process, and in tomatoes, most bHLH TFs were overexpressed in flower and leaf AZs during the abscission process (Sundaresan et al., 2016). The B3 superfamily of TFs has four subgroups, and these members have diverse functions in plant biological processes (Du et al., 2022), but which one is involved in plant organ shedding remains unclear. ERF and MYB TFs act as critical components in regulating flower and leaf abscission (Sundaresan et al., 2016). In cassava and tomato, the expression levels of some *ERF* and *MYB* genes are higher in the stages of leaf abscission (Liao et al., 2016a and Sundaresan et al., 2016; Liao et al., 2016c). Some *NAC* genes in AZs were upregulated in the TDZ-induced leaf abscission process (Li et al., 2022). ARFs are TFs that bind to auxin response elements in the promoters of early auxin response genes and play central roles in the plant organ abscission process (Guan et al., 2014). In the abscission process, the number of downregulated *ARF* genes exceeded that of upregulated *ARF* genes. Auxin delays abscission by upregulating more *ARF* genes, while ethylene accelerates abscission by downregulating more *ARF* genes (Guan et al., 2014). Therefore, these TFs involved in abscission might be the key players in the CYC-enhanced activity of TDZ at low temperatures. At the same time, the specific functions of the TFs in this process need to be studied further.

## 5 Conclusion

**Figure 13:**
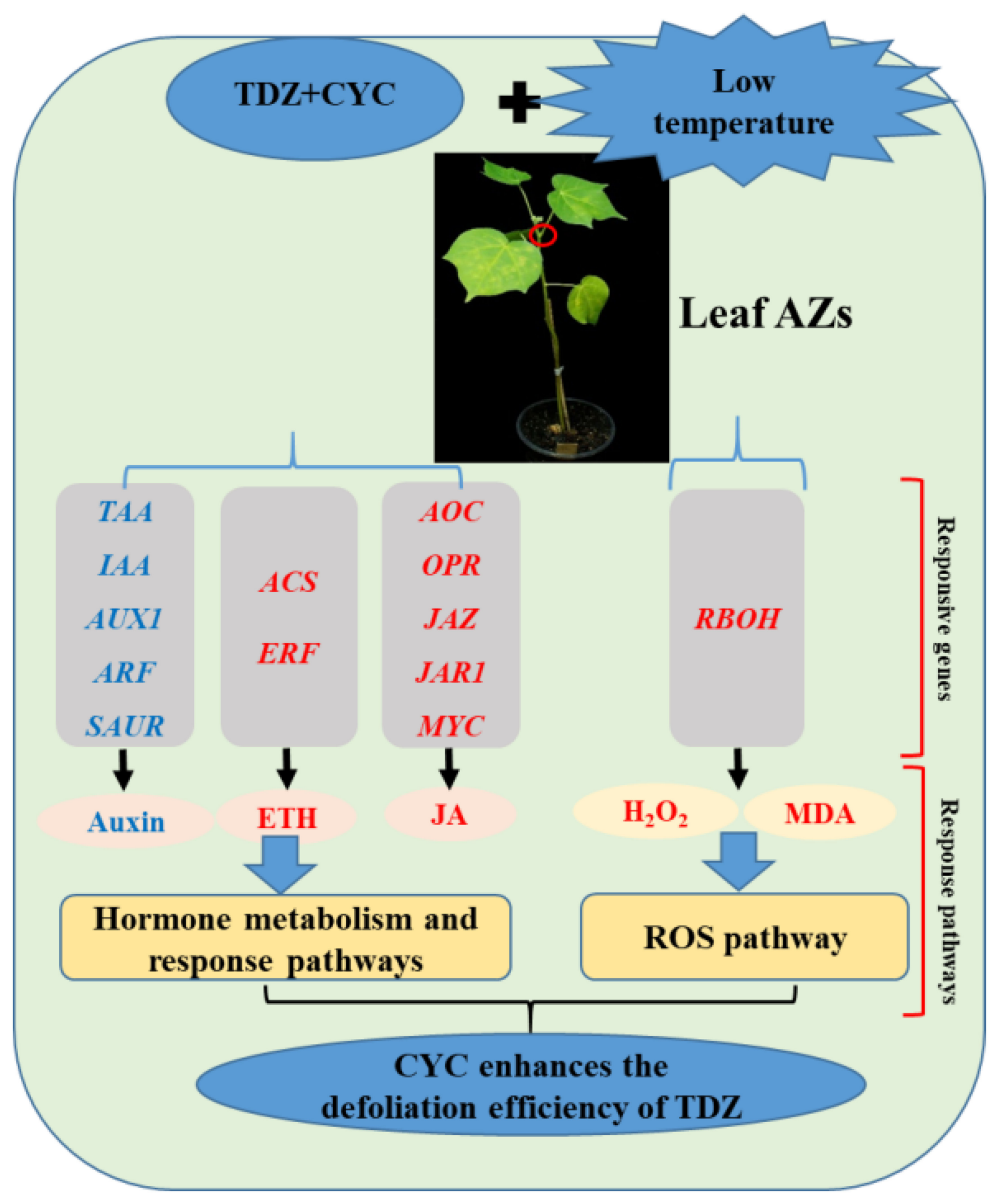
Schematic diagram of the mechanism by which CYC enhances the TDZ defoliation efficiency in cotton. Genes and metabolites in the plant hormone metabolism and response pathways and ROS pathway regulated by TDZ combined with CYC at low temperatures are listed. Red and blue fonts indicate upregulated and downregulated genes and metabolites, respectively.

This study clarified the mechanisms of the CYC-enhanced defoliation effect of TDZ at low temperatures through transcriptome analysis. The results showed that CYC enhanced the TDZ defoliation efficiency in cotton at low temperatures by regulating hormone (auxin, ethylene, and JA) related gene expression, including those that encode for biosynthesis, transport, signaling and response factor, and affecting ROS homeostasis. Key pathways, major responsive genes, and key TFs in cotton AZs were determined, and a regulatory mechanism model of CYC-enhanced TDZ defoliation efficiency at low temperatures was constructed (**Figure 13**). The results of this study not only contribute to understanding the CYC regulatory mechanism improving TDZ defoliation efficiency at low temperatures but also provide a theoretical basis for creating low-temperature resistant defoliants in cotton.

## Data availability statement

The original contributions presented in the study are included in the article/**Supplementary Material**, further inquiries can be directed to the corresponding author.

## Author contributions

HS: Conceptualization, Data curation, Formal analysis, Investigation, Methodology, Writing – original draft, Writing – review & editing. SS: Investigation, Writing – review & editing. XW: Investigation, Writing – review & editing. JC: Writing – review & editing. CY: Data curation, Writing – review & editing. GZ: Data curation, Writing – review & editing. HH: Writing – review & editing. ZL: Writing – review & editing. TL: Writing – review & editing. RL: Funding acquisition, Writing – review & editing, Conceptualization, Formal analysis.
