# Supplementary figures and images for: Thidiazuron combined with cyclanilide modulates hormone pathways and ROS systems in cotton, increasing defoliation at low temperatures
Source: Front Plant Sci. 2024 Apr 3;15:1333816. doi: 10.3389/fpls.2024.1333816 (PMC11021790; doi:10.3389/fpls.2024.1333816)

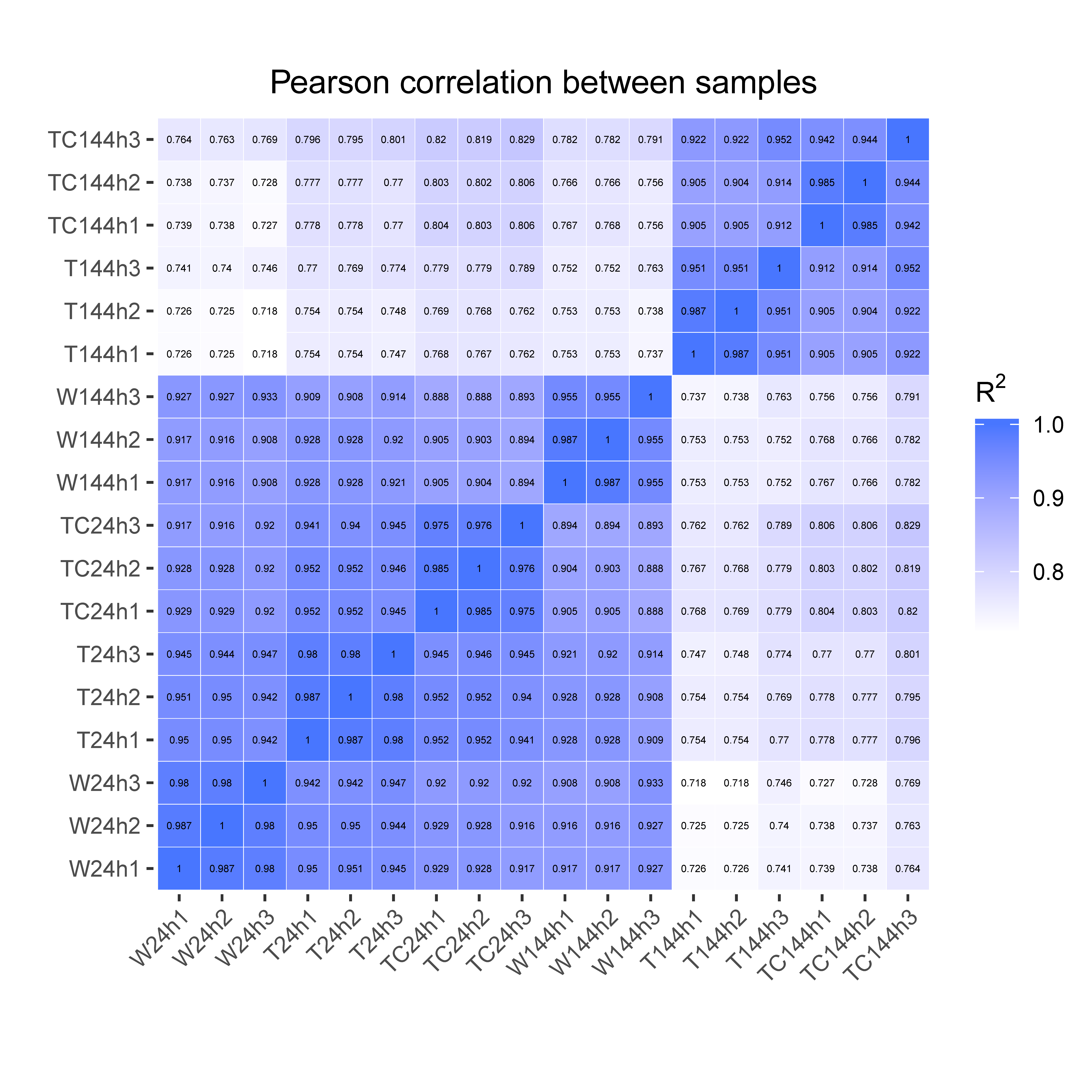

Supplement: Supplementary file 2 [file Image_2.tiff]
