# Supplementary material for: Thidiazuron combined with cyclanilide modulates hormone pathways and ROS systems in cotton, increasing defoliation at low temperatures
Source: Front Plant Sci. 2024 Apr 3;15:1333816. doi: 10.3389/fpls.2024.1333816 (PMC11021790; doi:10.3389/fpls.2024.1333816)
Supplement: Supplementary file 5 [file Table_1.docx]

**Supplemental TABLE 1** List of primers used in this study

| **Genes** |  | **Prime sequence (5’-3’)** |
| --- | --- | --- |
| *IAA9(Gh_A05G3764)* | F | CCAGAAGGTGGTAAAGGAC |
|  | R | AAACGATCTAATAGGAGGC |
| *ARF3(Gh_A05G1337)* | F | TGTCCTCACCGTCTTCAGT |
|  | R | GCTTCCTAGATACCTCCTAC |
| *AOC4(Gh_A08G0314)* | F | GCCAGACCCACCAGTAATA |
|  | R | TTTGGAGAAACGGATAGGA |
| *ERF1B(Gh_Sca115107G01)* | F | GGGAAATTCAGGATAGCGG |
|  | R | GGAGATAAGGGATTCAACGAG |
| *ACS(Gh_D12G2746)* | F | GGGTGATTGAGGTATGGGAGA |
|  | R | TGCATAAGCACAACGAGGC |
