# Supplementary material for: Thidiazuron combined with cyclanilide modulates hormone pathways and ROS systems in cotton, increasing defoliation at low temperatures
Source: Front Plant Sci. 2024 Apr 3;15:1333816. doi: 10.3389/fpls.2024.1333816 (PMC11021790; doi:10.3389/fpls.2024.1333816)
Supplement: Supplementary file 7 [file Table_3.docx]

**Supplemental files**

**Supplemental FIGURE 1** Analysis of all cDNA libraries (A) Principal component analysis (PCA). (B) Pearson correlation analysis.

**Supplemental FIGURE 2** The numbers of DEGs related to auxin, ethylene, jasmonic acid response pathways at 144 h post treatment. The DEGs are controlled by q-value < 0.05 and |log_2_FC| ≥ 1. Up represents upregulated DEGs. Down represents downregulated DEGs. T vs. W represents DEGs induced by the TDZ treatment compared with the control. TC vs. W represents DEGs induced by the TDZ+CYC treatment compared with the control. Black digits indicate the numbers of DEGs.

**Supplemental FIGURE 3** The expression levels of selected genes by qRT-PCR and RNAseq. Different lowercase letters in the same time point indicate significant differences at the 0.05 level, ns indicate no significant difference at the 0.05 level. W represents the control treatment. T represents TDZ treatment. TC represents TDZ+CYC treatment.

**Supplemental TABLE 1** List of primers used in this study

**Supplemental TABLE 2** The information of all transcripts in this study
